# Supplementary material for: Allosteric regulation and crystallographic fragment screening of SARS-CoV-2 NSP15 endoribonuclease
Source: Nucleic Acids Res. 2023 Apr 28;51(10):5255–70. doi: 10.1093/nar/gkad314 (PMC10250223; doi:10.1093/nar/gkad314)
Supplement: gkad314_Supplemental_Files [file gkad314_supplemental_files.zip › Supplementary datasets.zip!/224/Refine_9/01-BUSTER/Cycle-5/shell.01/R_Rfree.html]

# Rxpct factors in resolution bins at cycle 0


|  |  |  |  |  |  |  |  |  |
| --- | --- | --- | --- | --- | --- | --- | --- | --- |
|  | **<d> (Å)**  Rxpct\_Work | Rxpct\_Free | Rxpct\_All | |  |  |  |  |
|  | 15.0574 | 0.3701 | 0.4972 | 0.3745 |  |
|  | 8.6934 | 0.2294 | 0.2476 | 0.2304 |  |
|  | 6.7339 | 0.2122 | 0.2478 | 0.2142 |  |
|  | 5.6912 | 0.1869 | 0.2341 | 0.1885 |  |
|  | 5.0191 | 0.1497 | 0.2086 | 0.1533 |  |
|  | 4.5400 | 0.1356 | 0.1730 | 0.1375 |  |
|  | 4.1762 | 0.1411 | 0.1761 | 0.1431 |  |
|  | 3.8878 | 0.1495 | 0.2093 | 0.1521 |  |
|  | 3.6520 | 0.1580 | 0.2194 | 0.1609 |  |
|  | 3.4544 | 0.1611 | 0.2027 | 0.1635 |  |
|  | 3.2858 | 0.1734 | 0.2199 | 0.1761 |  |
|  | 3.1397 | 0.1785 | 0.2157 | 0.1802 |  |
|  | 3.0115 | 0.1847 | 0.2241 | 0.1864 |  |
|  | 2.8978 | 0.1983 | 0.2167 | 0.1992 |  |
|  | 2.7961 | 0.2026 | 0.2485 | 0.2047 |  |
|  | 2.7044 | 0.2086 | 0.2431 | 0.2103 |  |
|  | 2.6212 | 0.2096 | 0.2475 | 0.2118 |  |
|  | 2.5452 | 0.2268 | 0.2673 | 0.2291 |  |
|  | 2.4754 | 0.2362 | 0.2959 | 0.2396 |  |
|  | 2.4111 | 0.2594 | 0.3049 | 0.2614 |  |
|  | **Overall** | 0.1842 | 0.2262 | 0.1864 |  |

# Rxpct factors in resolution bins at cycle 1


|  |  |  |  |  |  |  |  |  |
| --- | --- | --- | --- | --- | --- | --- | --- | --- |
|  | **<d> (Å)**  Rxpct\_Work | Rxpct\_Free | Rxpct\_All | |  |  |  |  |
|  | 15.0574 | 0.3701 | 0.4972 | 0.3745 |  |
|  | 8.6934 | 0.2294 | 0.2476 | 0.2304 |  |
|  | 6.7339 | 0.2121 | 0.2478 | 0.2142 |  |
|  | 5.6912 | 0.1868 | 0.2340 | 0.1884 |  |
|  | 5.0191 | 0.1496 | 0.2086 | 0.1532 |  |
|  | 4.5400 | 0.1356 | 0.1730 | 0.1375 |  |
|  | 4.1762 | 0.1410 | 0.1761 | 0.1430 |  |
|  | 3.8878 | 0.1494 | 0.2093 | 0.1521 |  |
|  | 3.6520 | 0.1580 | 0.2194 | 0.1609 |  |
|  | 3.4544 | 0.1611 | 0.2026 | 0.1635 |  |
|  | 3.2858 | 0.1734 | 0.2199 | 0.1760 |  |
|  | 3.1397 | 0.1785 | 0.2157 | 0.1802 |  |
|  | 3.0115 | 0.1847 | 0.2241 | 0.1864 |  |
|  | 2.8978 | 0.1983 | 0.2167 | 0.1992 |  |
|  | 2.7961 | 0.2026 | 0.2485 | 0.2047 |  |
|  | 2.7044 | 0.2085 | 0.2430 | 0.2103 |  |
|  | 2.6212 | 0.2096 | 0.2475 | 0.2118 |  |
|  | 2.5452 | 0.2268 | 0.2675 | 0.2291 |  |
|  | 2.4754 | 0.2361 | 0.2958 | 0.2396 |  |
|  | 2.4111 | 0.2593 | 0.3050 | 0.2613 |  |
|  | **Overall** | 0.1842 | 0.2262 | 0.1863 |  |

# Rxpct factors in resolution bins at cycle 2


|  |  |  |  |  |  |  |  |  |
| --- | --- | --- | --- | --- | --- | --- | --- | --- |
|  | **<d> (Å)**  Rxpct\_Work | Rxpct\_Free | Rxpct\_All | |  |  |  |  |
|  | 15.0574 | 0.3700 | 0.4971 | 0.3744 |  |
|  | 8.6934 | 0.2293 | 0.2477 | 0.2304 |  |
|  | 6.7339 | 0.2119 | 0.2478 | 0.2140 |  |
|  | 5.6912 | 0.1866 | 0.2339 | 0.1881 |  |
|  | 5.0191 | 0.1494 | 0.2084 | 0.1530 |  |
|  | 4.5400 | 0.1353 | 0.1729 | 0.1373 |  |
|  | 4.1762 | 0.1408 | 0.1761 | 0.1428 |  |
|  | 3.8878 | 0.1493 | 0.2092 | 0.1519 |  |
|  | 3.6520 | 0.1579 | 0.2195 | 0.1608 |  |
|  | 3.4544 | 0.1610 | 0.2025 | 0.1634 |  |
|  | 3.2858 | 0.1733 | 0.2198 | 0.1760 |  |
|  | 3.1397 | 0.1785 | 0.2158 | 0.1801 |  |
|  | 3.0115 | 0.1847 | 0.2241 | 0.1864 |  |
|  | 2.8978 | 0.1982 | 0.2168 | 0.1992 |  |
|  | 2.7961 | 0.2025 | 0.2487 | 0.2047 |  |
|  | 2.7044 | 0.2085 | 0.2430 | 0.2102 |  |
|  | 2.6212 | 0.2095 | 0.2475 | 0.2118 |  |
|  | 2.5452 | 0.2267 | 0.2677 | 0.2290 |  |
|  | 2.4754 | 0.2360 | 0.2957 | 0.2395 |  |
|  | 2.4111 | 0.2592 | 0.3052 | 0.2612 |  |
|  | **Overall** | 0.1841 | 0.2262 | 0.1862 |  |

# Rxpct factors in resolution bins at cycle 3


|  |  |  |  |  |  |  |  |  |
| --- | --- | --- | --- | --- | --- | --- | --- | --- |
|  | **<d> (Å)**  Rxpct\_Work | Rxpct\_Free | Rxpct\_All | |  |  |  |  |
|  | 15.0574 | 0.3690 | 0.4943 | 0.3734 |  |
|  | 8.6934 | 0.2251 | 0.2563 | 0.2269 |  |
|  | 6.7339 | 0.2076 | 0.2565 | 0.2104 |  |
|  | 5.6912 | 0.1812 | 0.2291 | 0.1828 |  |
|  | 5.0191 | 0.1442 | 0.2067 | 0.1480 |  |
|  | 4.5400 | 0.1319 | 0.1778 | 0.1342 |  |
|  | 4.1762 | 0.1370 | 0.1778 | 0.1393 |  |
|  | 3.8878 | 0.1461 | 0.2134 | 0.1491 |  |
|  | 3.6520 | 0.1566 | 0.2179 | 0.1595 |  |
|  | 3.4544 | 0.1593 | 0.2040 | 0.1619 |  |
|  | 3.2858 | 0.1718 | 0.2189 | 0.1745 |  |
|  | 3.1397 | 0.1792 | 0.2164 | 0.1808 |  |
|  | 3.0115 | 0.1850 | 0.2267 | 0.1868 |  |
|  | 2.8978 | 0.1973 | 0.2162 | 0.1983 |  |
|  | 2.7961 | 0.2030 | 0.2543 | 0.2053 |  |
|  | 2.7044 | 0.2083 | 0.2470 | 0.2103 |  |
|  | 2.6212 | 0.2109 | 0.2486 | 0.2131 |  |
|  | 2.5452 | 0.2278 | 0.2713 | 0.2302 |  |
|  | 2.4754 | 0.2374 | 0.2919 | 0.2405 |  |
|  | 2.4111 | 0.2604 | 0.3162 | 0.2628 |  |
|  | **Overall** | 0.1819 | 0.2282 | 0.1842 |  |
